# Preventing major adverse cardiovascular events by SGLT-2 inhibition in patients with type 2 diabetes: the role of kidney

**DOI:** 10.1186/s12933-020-01010-x

**Published:** 2020-03-19

**Authors:** Dario Giugliano, Luca De Nicola, Maria Ida Maiorino, Giuseppe Bellastella, Carlo Garofalo, Paolo Chiodini, Antonio Ceriello, Katherine Esposito

**Affiliations:** 1 Division of Endocrinology and Metabolic Diseases, Department of Advanced Medical and Surgical Sciences, Università della Campania L. Vanvitelli, Naples, Italy; 2 Nephrology Unit, Department of Advanced Medical and Surgical Sciences, Università della Campania L. Vanvitelli, Naples, Italy; 3 Diabetes Unit, Department of Advanced Medical and Surgical Sciences, Università della Campania L. Vanvitelli, Naples, Italy; 4 Medical Statistics Unit, Università della Campania Luigi Vanvitelli, Naples, Italy; 5 Department of Cardiovascular and Metabolic Diseases, IRCCS MultiMedica, Sesto San Giovanni, MI Italy

**Keywords:** SGLT-2 inhibitors, Type 2 diabetes, MACE, Diabetic kidney disease, Statin therapy

## Abstract

Cardiovascular outcome trials (CVOTs) have demonstrated a significant reduction of major adverse cardiovascular events (MACE) in patients with type 2 diabetes (T2D) treated by SGLT-2 inhibitors. This holds true in the presence of background therapy with statins in most patients. Noteworthy, this SGLT-2 inhibitors effect is unique because, at variance with other components of cardiorenal protection, MACE prevention does not appear to be a class effect. Here, we present meta-analysis of the four key CVOTs indicating a major role of renal function in determining the extent of MACE prevention, with the benefit increasing in more severe kidney disease, that is, a high-risk condition where effectiveness of the traditional approach with statins is reduced.

The recent cardiovascular outcome trials (CVOTS) [1–4] testing the cardiorenal effects of SGLT-2 inhibitors in patients with type 2 diabetes mellitus (T2D) have demonstrated a significant reduction of major adverse cardiovascular events (MACE). The clinical significance of this effect is even more relevant when considering that SGLT-2 inhibitors were added on the top of optimal therapy, including renin-angiotensin system (RAS) inhibitors and statins in the vast majority of cases.

The risk reduction for MACE ranges from 7% of DECLARE trial (not significant) to 20% of CREDENCE trial (significant), with intermediate and significant reduction (14%) in both EMPA-REG OUTCOME and CANVAS trials. As the cardiorenal protection by SGLT-2 inhibition is considered a class effect [5, 6], the reasons for this divergence is not readily apparent, even because the major risk factors for MACE (age, smoking, body weight, blood pressure and lipids) were on average similarly controlled in the four trials, and treatment with RAS inhibitors and statins were comparable (Table 1). It has been suggested that the observed differences are inherent to the population studied; in the DECLARE trial, patients were globally (and relatively) healthier at baseline, which reduced the power to detect differences between the two arms of the study. In particular, most patients in DECLARE had less atherosclerotic disease and more preserved renal function. On the other hand, the extent of risk reduction for MACE was definitely greater in the CREDENCE trial where atherosclerotic disease was prevalent and renal function severely impaired.

**Table 1:** Main basal risk factors for MACE in the cardiovascular outcome trials testing the effect of SGLT2 inhibition in type 2 diabetes

Trial Sample size	SGLT2-I	Age, years	Smokers, % pts	BMI, kg/m^2^	Systolic BP, mmHg	HbA1c, %	LDL-C, mg/dL	ACVD, % pts	eGFR, mL/min/1.73 m^2^	eGFR < 60, % pts	Anti-RAS, % pts	Statin, % pts
EMPA-REG *n *= 7021	Empa	63 ± 9	13.0	31 ± 5	135 ± 17	8.1 ± 0.9	86 ± 36	89	74 ± 22	26.0	81.0	77.4
CANVAS *n *= 10,142	Cana	63 ± 8	17.8	32 ± 6	137 ± 16	8.2 ± 0.9	89 ± 35	72	77 ± 21	25.0	80.2	74.7
DECLARE *n *= 17,160	Dapa	64 ± 7	14.5	32 ± 6	135 ± 15	8.3 ± 1.2	89 ± 35	41	85 ± 16	7.0	81.3	74.9
CREDENCE *n *= 4401	Cana	63 ± 9	14.5	31 ± 6	140 ± 16	8.3 ± 1.3	96 ± 41	69	56 ± 18	59.8	99.9	69.8

MACE: major adverse cardiovascular evets, that is, cardiovascular death, myocardial infarction, or ischemic stroke; BP: blood pressure; LDL-C: LDL cholesterol; ACVD: atherosclerotic cardiovascular disease; Anti-RAS: inhibitors of renin angiotensin system; CVD: cardiovascular disease; eGFR: estimated glomerular filtration rate

## MACE risk in diabetic kidney disease

In order to assess whether the benefits exerted by SGLT-2 inhibitors on MACE may be positively associated to the greater renal impairment at baseline, we did a meta-analysis of the four CVOTs [1–4] with SGLT-2 inhibitors on MACE risk, as compared with placebo. Hazard ratios (HRs) and 95% confidence intervals (CIs) for efficacy outcomes were synthesized. Heterogeneity among studies was evaluated using the Cochran’s Q test, with P values of less than 0.10 representing significant heterogeneity. We did an additional sensitivity analysis to assess the effects of treatment in participants with eGFR lower than 60 mL/min per 1.73 m^2^ and those with an eGFR of 60 mL/min per 1.73 m^2^ or greater. When required, effect estimates for subgroups within the same study (e.g., eGFR 30 to < 45 mL/min per 1.73 m^2^ and 45 to < 60 mL/min per 1.73 m^2^, or eGFR > 60 to 90 mL/min per 1.73 m^2^ and > 90 mL/min per 1.73 m^2^) were merged by use of a fixed-effects model. We limited the evaluation to MACE in order to minimize the statistical impact of post hoc analyses. Pooled summary estimates were calculated according to the random effects model, using the empirical Bayes method that, in Stata software, corresponds to the Paule–Mandel method [7]. In subgroup analysis, p-heterogeneity value lower than 0.1 was considered to reflect a high likelihood of difference beyond that expected by chance [8]. All analyses were done with Stata, version 16.0 (Stata Corp., College Station, TX).

**Fig. 1:**
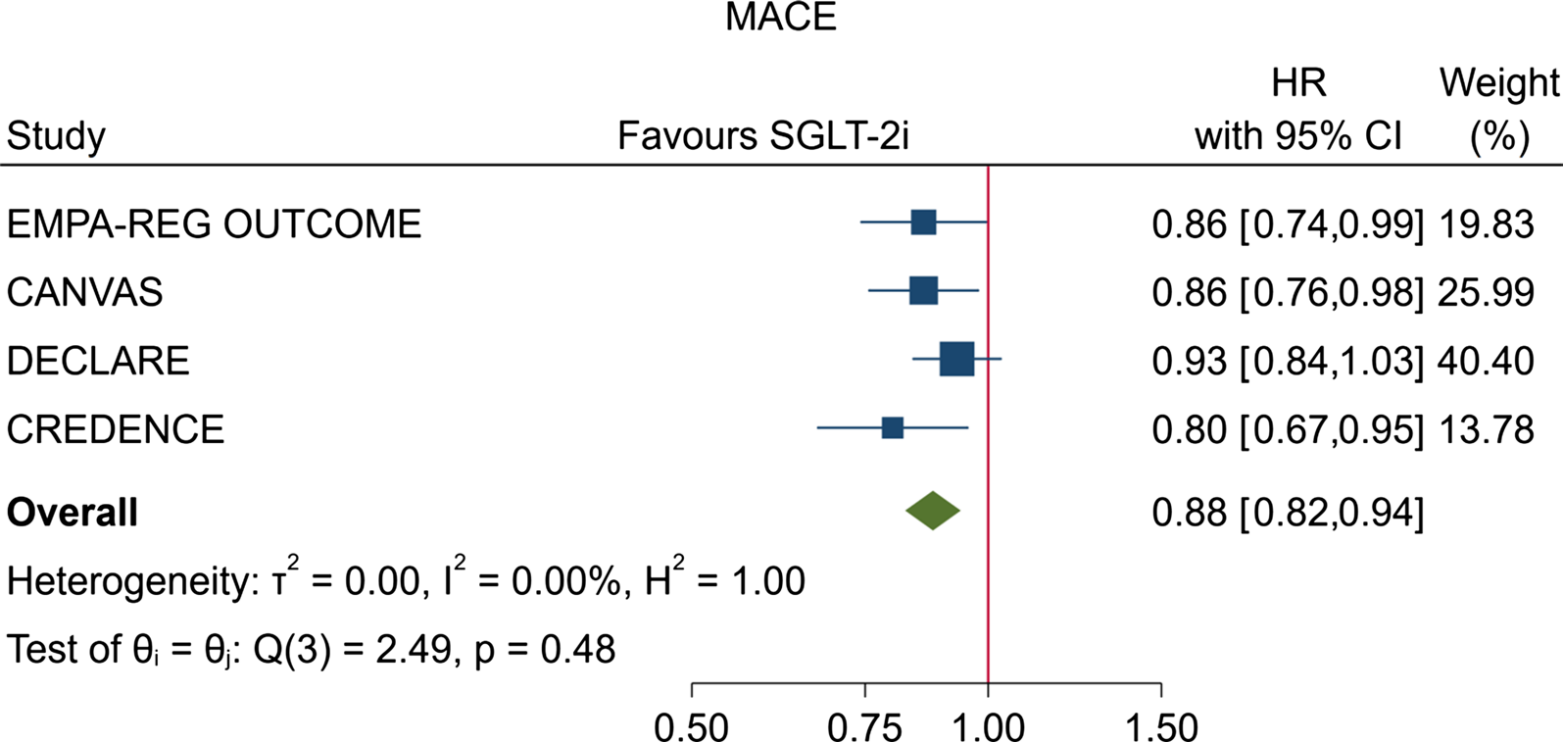
Effect of SGLT-2 inhibitors on risk of MACE in the four CVOTs with gliflozins

**Fig. 2:**
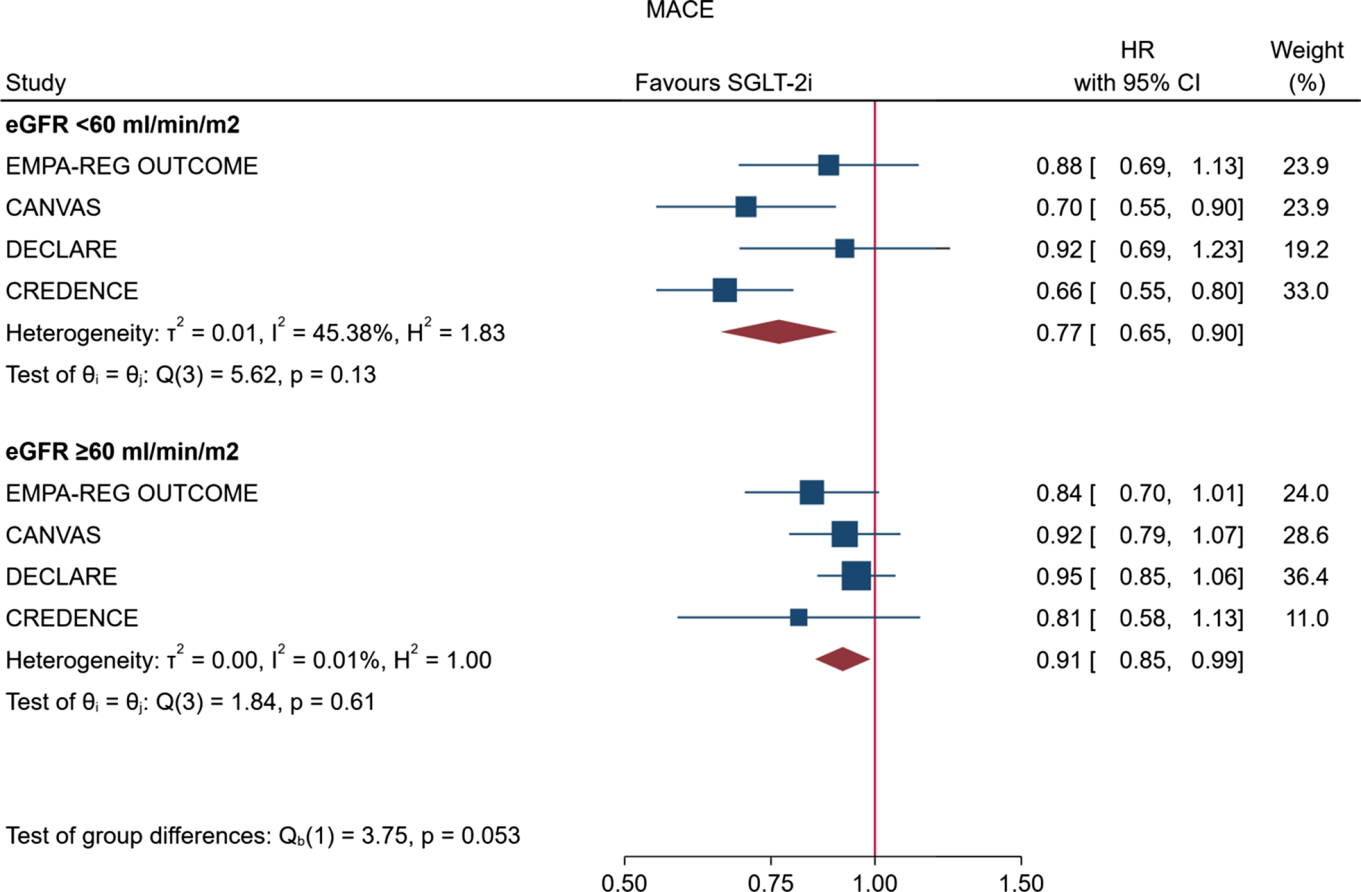
Random-effect meta-analysis describing the effect of the four CVOTs with gliflozins on the primary endpoint (MACE) according to the renal function of patients with T2D

Our meta-analysis included data for a total of 38 724 randomly assigned participants from six continents. The proportion of participants with an eGFR less than 60 mL/min per 1.73 m^2^ ranged from 7.4% in DECLARE–TIMI 58 to 59.8% in CREDENCE, and the proportion of participants taking anti-RAS therapy ranged from 80% (CANVAS) to almost 100% (CREDENCE) (Table 1). In the overall analysis, risk of MACE was reduced by 12% (HR, 0.88; 95% CI 0.82–0.94; P < 0.001) compared with placebo, with null heterogeneity among trials (I^2^ = 0%) (Fig. 1). Most patients had preserved kidney function, with 7754 participants (20%) with baseline eGFR < 60 mL/min per 1.73 m^2^. There was evidence that patients with reduced kidney function achieved greater proportional risk reductions for MACE (HR, 0.77; 95% CI 0.65–0.90) than patients with preserved kidney function (HR, 0.91; 95% CI 0.85–0.99) (P for heterogeneity between subgroups = 0.053) (Fig. 2).

## Renal function and MACE

The relationship between renal function and MACE is well established. The ADVANCE study evidenced in more than 10,000 patients with T2D aged ≥ 55 years that higher albuminuria and lower eGFR levels predict increased risk of CV events, including CV death, non-fatal myocardial infarction and non-fatal stroke [9]. The independent role of kidney disease in dictating the CV prognosis has been further supported by a large meta-analysis of CKD Consortium, in more than one million subjects, evidencing that hazards for CV mortality at a given eGFR or ACR were 1.2 to 1.9 times higher in diabetic vs non diabetic individuals across the whole spectrum of eGFR and ACR strata. However, no interaction was disclosed on CV risk between CKD severity and diabetic status, supporting the idea of the independent role of kidney disease as CV risk modifier [10]. This concept has been reinforced by a similarly large study from the Alberta Kidney Disease Network showing that in patients with no previous history of heart diseases, the incidence of acute myocardium infarction did not differ in people with diabetes and no CKD versus those with CKD stage 1–4 and no diabetes [11]. The study also demonstrated that in more advanced CKD without diabetes (eGFR < 45 mL/min per 1.73 m^2^ and severe proteinuria), the risk of coronary events actually overcame that observed in diabetic patients without CKD. On the other hand, CKD is prevalent in coronary heart disease: the EUROASPIRE IV study, that evaluated patients with coronary heart disease followed in cardiology setting from 24 European countries, found that as many as one-third population had CKD (eGFR < 60 mL/min/1.73 m^2^ in 17.3% while albuminuria with normal eGFR was detected in an additional 12%) [12].

Overall, these findings have allowed to identify CKD as a “coronary heart disease risk equivalent” as it was recognized for T2D. With the notable difference that T2D may no longer be a risk equivalent for coronary heart disease if all risk factors (glycemia, blood pressure, LDL-cholesterol, body weight, smoking) are being controlled by treatment (drug and/or lifestyle) [13, 14]; this may not the case for CKD as its current therapeutic armamentarium does not restore to normal albuminuria, eGFR, or both. CKD also acts as major predisposing risk factor also for stroke and peripheral artery disease [15, 16]. Indeed, impaired renal function and abnormal albuminuria can per se promote endothelial dysfunction and accelerate atherosclerosis, independently from hypertension and diabetes, and this phenomenon strongly modifies the fate of CKD patients so that a vast proportion of CKD patients are more likely to die prematurely due to CV disease than to survive long enough to reach end-stage kidney disease (ESKD) [17, 18].

## SGLT-2 inhibition as anti-MACE therapy in diabetic kidney disease (DKD)

The four CVOT have provided a strong argument favoring the use of SGLT-2 inhibitors for prevention of MACE in diabetic patients with or without DKD. These trial-derived data have been confirmed in the large, multinational CVD-REAL study comparing CV outcome in patients initiated on SGLT2-inhibitors versus other glucose-lowering drugs [19]. Indeed, in this propensity-matching study (n = 235,034 patients in either group), SGLT-2 inhibitors reduced the risk of myocardial infarction and stroke by 19% and 32%, respectively: these beneficial effects were obtained in a low-risk population because CVD-REAL patients had a positive history of CV disease in less than 30% cases and CKD prevalence was irrelevant (< 2%), and most patients were under RAS inhibitors (55%) and statins (65%). The findings from this large real-world study must be integrated with those of CVOT. Indeed, MACE prevention by means of SGLT-2 inhibition, which was more evident in the higher risk CVOT, emerges also in low-risk patients when the sample examined is very large.

The beneficial effects of SGLT-2 inhibitors on MACE are probably multifactorial [20, 21]: they are thought to produce benefits on cardiovascular system through their ability to reduce myocardial inflammation, oxidative stress, apoptosis, mitochondrial dysfunction, ionic dyshomeostasis, preload, cardiac stretch, blood pressure and after load, and increased natriuresis. Moreover, it is possible to hypothesize that the remarkable nephroprotection may play a major role. Prevention of eGFR decline may indeed protect from MACE not only directly but also indirectly by decreasing progressive decline of renal function. Accordingly, the recent results of CVD-REAL 3 study in over 70,000 diabetic patients, i.e., the first real-world data analysis on the effects of SGLT-2 inhibitors on renal outcomes, are of great interest [22]. Investigators demonstrated that initiation of SGLT-2 inhibitors allowed 51% reduction of the composite outcome of a 50% decline in eGFR or ESKD (3.0 vs 6.3 events per 10,000 patient-years). The importance of these results is in the low-risk population under study; patients had on average an eGFR 91 mL/min/1.73 m^2^ with only less than 10% of whole cohort being classified with overt CKD (stage 3 or higher).

## Conclusions

The data herein presented provide robust evidence of cardiovascular (MACE) benefits with SGLT-2 inhibitors in patients with T2D, with significant evidence that effects may be even greater in patients with DKD. Statin therapy, while not substantially modifying renal risk, leaves a substantial portion of CKD patients, especially in those with more advanced stages of disease, at high risk of CV events [23, 24]. These findings indicate that a broad range of patients with T2D are likely to achieve important benefits from use of this drug class [25] and should encourage physicians taking care of diabetic patients to an early use of SGLT-2 inhibitors in order to slow down progression of diabetic kidney disease and prevent MACE.

## Data Availability

Not applicable

## Abbreviations

SGLT-2: Sodium–glucose transporter-2
T2D: Type 2 diabetes
DKD: Diabetic kidney disease
CVOTs: Cardiovascular outcome trials
EMPAREG-OUTCOME: Empagliflozin cardiovascular outcome event trial in type 2 diabetes mellitus patients-removing excess glucose randomized double-blind controlled
CANVAS: CANagliflozin CardioVascular Assessment Study
DECLARE: Dapagliflozin Effect on CardiovascuLAR Events randomized double-blind controlled trial
CREDENCE: Canagliflozin and Renal Events in Diabetes with Established Nephropathy Clinical Evaluation randomized double-blind controlled trial
ADVANCE: Action in Diabetes and Vascular disease: preterAx and diamicroN-MR Controlled Evaluation
CVD-REAL: Comparative effectiveness of cardiovascular outcomes in new users of SGLT-2 inhibitors
